# Association between cardiometabolic index and kidney stone from NHANES: a population-based study

**DOI:** 10.3389/fendo.2024.1408781

**Published:** 2024-10-09

**Authors:** Qianqian Wang, Zhaoxiang Wang, Zaixiang Tang, Can Liu, Ying Pan, Shao Zhong

**Affiliations:** ^1^ Department of Endocrinology, Affiliated Kunshan Hospital of Jiangsu University, Kunshan, Jiangsu, China; ^2^ Department of Biostatistics, School of Public Health, Jiangsu Key Laboratory of Preventive and Translational Medicine for Geriatric Diseases, Ministry of Education (MOE) Key Laboratory of Geriatric Diseases and Immunology, Suzhou Medical College of Soochow University, Suzhou, Jiangsu, China

**Keywords:** NHANES, cardiometabolic index, kidney stone, population-based study, linear relationship

## Abstract

**Purpose:**

The Cardiometabolic Index (CMI) is a novel marker of visceral obesity and dyslipidemia. Our study aimed to explore the association between CMI and kidney stones among US adults.

**Methods:**

This cross-sectional study was conducted among adults with complete records of CMI and kidney stones information from the 2011 to 2018 National Health and Nutrition Examination Survey (NHANES). Inverse probability treatment weighting (IPTW) was used to balance the baseline characteristics of the study population. The independent relationship between CMI and kidney stones was evaluated using IPTW-adjusted multivariate logistic regression, restricted cubic splines (RCS), and subgroup analysis.

**Results:**

A total of 9,177 participants, with an average CMI of 0.72 (0.99), were included in this study. The IPTW-adjusted logistic regression revealed that CMI was an independent risk factor for kidney stones. The adjusted odds ratio (OR) for kidney stones were 1.39 (95% CI: 1.24 – 1.56, *P* < 0.001) for the second CMI tertile and 1.31 (95% CI: 1.17 – 1.47, *P* < 0.001) for the third CMI tertile, compared with the first CMI tertile. A linear relationship between CMI levels and kidney stone risk was observed in the RCS analysis. Subgroup analysis showed that the association between CMI levels and kidney stone risk remained stable across groups.

**Conclusions:**

A positive association between CMI level and the risk of kidney stones was observed among US adults in our study. Further large-scale prospective studies are needed to validate our findings.

## 1 Introduction

Kidney stone disease is a highly prevalent urinary system disorder that affects approximately 10% of the population (1). Kidney stones typically result in acute attacks characterized by renal colic, urinary obstruction, urinary tract infections, and, in severe cases, kidney failure or life-threatening conditions (2). Despite advancements in minimally invasive and non-invasive lithotripsy techniques, up to 50% of patients post-surgery still experience kidney stone recurrence, significantly increasing both physiological and economic burdens (3). The rising incidence and recurrent nature of kidney stone disease contributed to substantial healthcare expenditures and diminished quality of life, underscoring the significance of identifying modifiable risk factors for early targeted interventions (4). It is important to identify individuals with high kidney stone risk using simple and effective diagnostic indicators (5, 6).

Emerging evidence underscored metabolic abnormalities as pivotal in the pathogenesis of kidney stones (7). Kidney stone disease may be a renal manifestation of systemic diseases such as metabolic syndrome rather than an isolated disorder caused by disturbances in urine composition (8, 9). Some studies revealed a correlation between obesity, lipid metabolic disturbances, and the occurrence of kidney stone (10, 11). Furthermore, the distribution of body fat, particularly the increase in visceral adiposity, was closely related to the metabolic risk factors for kidney stones (12, 13). Due to the complexity and systemic nature of kidney stones, multidimensional assessment is particularly important (14, 15). The Cardiometabolic Index (CMI) is a novel anthropometric index calculated by Triglyceride (TG)/High-density lipid cholesterol (HDL-c) × waist-to-height ratio (WHtR) (16). WHtR was deemed a more precise indicator of certain health risks compared to body mass index (BMI), due to its focus on the distribution of body fat (17). Additionally, the TG/HDL-c ratio became a commonly acknowledged indicator of lipid metabolism disorders (18). Integrating these two indices effectively, CMI was suggested as a more comprehensive assessment of abdominal obesity and dyslipidemia, thereby providing a more holistic approach to evaluating metabolic health (19). Several studies indicated that CMI was a promising indicator of diabetes, cardiovascular disease, metabolic syndrome, and renal dysfunction (20–23).

To our knowledge, the relationship between CMI level and kidney stone risk is still unclear. Therefore, our study aimed to systematically investigate the relationship between CMI and kidney stones based on the National Health and Nutrition Examination Survey (NHANES) database, potentially paving the way for novel preventative strategies and early screening methods for kidney stone disease.

## 2 Materials and methods

### 2.1 Data source

This population-based study utilized data from the NHANES, which aimed to assess the health and nutritional status of the US population. NHANES was a randomized, stratified, multi-stage survey design, providing a nationally representative sample with detailed demographic, socioeconomic, and health information (24). The study protocol for the NHANES database received approval from the Ethics Review Board of the National Center for Health Statistics. We included participants with complete data on kidney stones from 2011 to 2018. Participants below 20 years, those pregnant, and individuals without complete CMI data or questionnaire records about kidney stones were excluded. Adhering to these criteria, 9,177 patients were eligible for our study.

### 2.2 Exposure and outcome definitions

The CMI was calculated as the TG/HDL-c × WHtR (25). The primary outcome for our analysis was the participants’ response to the question “Have you or the sample person ever had a kidney stone?” during the medical questionnaire (26, 27). The participants who reported “yes” to the question were considered as having kidney stones, while those answering “no” were classified as not. The reliability of self-reported kidney stone conditions has been validated in prior studies (28–32).

### 2.3 Covariate definitions

Based on existing literature and clinical experience, confounding factors covering demographics, lifestyle habits, and health indicators were selected. Previous studies have confirmed these variables to have a significant association with kidney stones risk (33–38): demographic data (age, gender, race), annual household income, education level, physical activity level, smoking status, diabetes prevalence, hypertension prevalence, cardiovascular disease prevalence, BMI, alanine aminotransferase (ALT), aspartate aminotransferase (AST), gamma-glutamyl transferase (GGT), albumin, fasting plasma glucose (FPG), glycated hemoglobin A1c (HbA1c), total cholesterol (TC), low-density lipoprotein cholesterol (LDL-c), blood urea nitrogen (BUN), serum creatinine (Scr), serum uric acid (SUA), and estimated glomerular filtration rate (eGFR). BMI was categorized into three groups: < 25 kg/m^2^ (normal weight), 25 - 29.9 kg/m^2^ (overweight), and ≥ 30 kg/m^2^ (obese). eGFR was calculated according to the CKD Epidemiology Collaboration creatinine equation, which incorporates factors including age, gender, race, and Scr (39). Additionally, self-reported diabetes and hypertension were identified, and cardiovascular disease presence was assessed through self-reports of coronary artery disease, angina, heart attack, congestive heart failure, or stroke (40). Comprehensive details of study variables are publicly available in the NHANES database (https://www.cdc.gov/nchs/nhanes/).

### 2.4 Statistical analysis

All statistical analyses were conducted following the guidelines set forth by the Centers for Disease Control and Prevention, incorporating a complex, multistage cluster survey design (41). Continuous variables were expressed as mean with standard deviation, and categorical parameters were presented as proportions. Student’s t-test and chi-squared test were used to compare baseline variable differences. The Inverse probability of treatment weighting (IPTW) was used to control for confounders among the three exposure groups (according to the tertiles of CMI). Standardized mean difference (SMD) was used to assess the variation of CMI tertiles, with an SMD < 0.1 indicating a better equilibrium. To explore the relationship between CMI tertiles and the risk of kidney stones, odds ratio (OR) and IPTW-adjusted OR with 95% confidence interval (CI) were reported using Logistic Regression Models (Model 1, no covariate was adjusted; Model 2, adjusted for age, gender, and race; Model 3, further adjusted for annual household income, education level, physical activity level, smoking status, diabetes prevalence, hypertension prevalence, cardiovascular disease prevalence, BMI class, ALT, AST, GGT, albumin, TC, LDL-c, FPG, HbA1c, SUA, Scr, BUN and eGFR class). To investigate potential non-linear relationships, we employed a restricted cubic splines (RCS) analysis using the ‘rms’ R package (version 6.7.1) with four knots. Additionally, subgroup analyses were conducted using logistic regression models stratified by age (< 60/≥ 60 years), gender (female/male), BMI (normal weight/overweight/obese), diabetes (yes/no), hypertension (yes/no), cardiovascular disease (yes/no), and eGFR (< 60/60-90/≥ 90ml/min/1.73m^2^). All statistical analyses were performed using R software and the Empower software (http://www.empowerstats.com), with ‘dplyr’ package (version 1.1.4) for data manipulation and preprocessing, ‘ggplot2’ package (version 3.5.0) for plots and visualizations. A two-sided ***P*** value < 0.05 was considered statistically significant.

## 3 Results

### 3.1 Baseline characteristics of the study population

A total of 9,177 individuals were included (48.75% male) with a mean age of 49.63 years in this study. Among the total participants, 908 individuals (9.89%) had kidney stones, which corresponded with the estimated prevalence rates in the general population (3). Compared with those without kidney stones, participants with kidney stones tend to be male, older and have a higher BMI, waist circumference (WC), FPG, HbA1c, TG, smoking rates, diabetes prevalence, hypertension prevalence, and cardiovascular disease prevalence (*P* < 0.05) (**Table 1**). Conversely, HDL-c, albumin, and impaired kidney function (characterized by elevated SUA, Scr, BUN, and reduced eGFR), were evident in the kidney stone group (*P* < 0.05). Racial disparity was observed in kidney stones (*P* < 0.001). Specifically, the Non-Hispanic White group exhibited the highest risk of developing kidney stones, while the Non-Hispanic Black group demonstrated the lowest risk (*P* < 0.001). However, there were no significant differences in annual household income (*P* = 0.741), education level (*P* = 0.706), physical activity level (*P* = 0.257), height (*P* = 0.627), ALT (*P* = 0.081), AST (*P* = 0.15), GGT (*P* = 0.858), TC (*P* = 0.143), and LDL-c (*P* = 0.303). It was noted that CMI levels were higher in the kidney stone group than in the non-kidney stone group (0.71 ± 0.98 vs. 0.88 ± 1.08, *P* < 0.001).

**Table 1:** Baseline characteristics of the study population in NHANES from 2011 to 2018.

		Overall	No kidney stone	Kidney stone	*P*
n		9,177	8,269	908	
Age (mean (SD))		49.63 (17.40)	49.02 (17.43)	55.20 (16.07)	<0.001
Age class (%)	≥60	6121 (66.70)	5617 (91.77)	504 (8.23)	<0.001
	≥60	3056 (33.30)	2652 (86.78)	404 (13.22)	
Gender (%)	Female	4703 (51.25)	4277 (90.94)	426 (9.06)	0.006
	Male	4474 (48.75)	3992 (89.23)	482 (10.77)	
Race (%)	Mexican American	1241 (13.52)	1117 (90.01)	124 (9.99)	<0.001
	Non-Hispanic Black	1948 (21.23)	1842 (94.56)	106 (5.44)	
	Non-Hispanic White	3483 (37.95)	3027 (86.91)	456 (13.09)	
	Other Hispanic	991 (10.80)	887 (89.51)	104 (10.49)	
	Other Races	1514 (16.50)	1396 (92.21)	118 (7.79)	
Annual household income (%)	Above $20,000	6876 (79.62)	6191 (90.04)	685 (9.96)	0.741
	Under $20,000	1760 (20.38)	1580 (89.77)	180 (10.23)	
Education level (%)	Below high school	4037 (44.01)	3632 (89.97)	405 (10.03)	0.706
	Above high school	5135 (55.99)	4632 (90.20)	503 (9.80)	
Physical activity level (%)	Low	5372 (58.60)	4840 (90.10)	532 (9.90)	0.257
	Moderate	1945 (21.22)	1768 (90.90)	177 (9.10)	
	High	1851 (20.19)	1653 (89.30)	198 (10.70)	
Smoking status (%)	No	5192 (56.63)	4734 (91.18)	458 (8.82)	<0.001
	Yes	3977 (43.37)	3528 (88.71)	449 (11.29)	
Diabetes (%)	No	7902 (86.14)	7212 (91.27)	690 (8.73)	<0.001
	Yes	1271 (13.86)	1053 (82.85)	218 (17.15)	
Hypertension (%)	No	5777 (63.02)	5312 (91.95)	465 (8.05)	<0.001
	Yes	3390 (36.98)	2947 (86.93)	443 (13.07)	
Cardiovascular disease (%)	No	8177 (89.10)	7433 (90.90)	744 (9.10)	<0.001
	Yes	1000 (10.90)	836 (83.60)	164 (16.40)	
BMI		29.20 (6.96)	29.02 (6.92)	30.85 (7.08)	<0.001
BMI class (%)	Normal weight	2673 (29.16)	2508 (93.83)	165 (6.17)	<0.001
	Overweight	2965 (32.34)	2660 (89.71)	305 (10.29)	
	Obese	3530 (38.50)	3092 (87.59)	438 (12.41)	
Height (mean (SD))		166.98 (9.96)	167.00 (9.94)	166.83 (10.13)	0.627
WC (mean (SD))		99.61 (16.65)	99.01 (16.53)	105.08 (16.68)	<0.001
ALT (mean (SD))		24.38 (17.33)	24.42 (17.72)	24.02 (13.28)	0.081
AST (mean (SD))		24.66 (18.75)	24.75 (19.42)	23.80 (10.86)	0.15
GGT (mean (SD))		29.43 (38.20)	29.45 (38.86)	29.21 (31.62)	0.858
Albumin (mean (SD))		41.99 (3.51)	42.05 (3.53)	41.43 (3.32)	<0.001
BUN (mean (SD))		4.95 (2.10)	4.90 (2.05)	5.38 (2.46)	<0.001
Scr (mean (SD))		78.34 (38.10)	77.92 (36.14)	82.12 (52.51)	<0.001
SUA (mean (SD))		326.12 (85.89)	325.48 (85.52)	331.88 (89.04)	0.033
eGFR class (%)	<60	671 (7.34)	570 (84.95)	101 (15.05)	<0.001
	60~90	2788 (30.48)	2448 (87.80)	340 (12.20)	
	≥90	5687 (62.18)	5221 (91.81)	466 (8.19)	
FPG (mean (SD))		6.13 (2.02)	6.09 (1.99)	6.57 (2.24)	<0.001
HbA1c (mean (SD))		5.81 (1.14)	5.79 (1.13)	6.05 (1.23)	<0.001
HDL-c (mean (SD))		1.40 (0.42)	1.41 (0.42)	1.31 (0.38)	<0.001
TG (mean (SD))		1.35 (1.21)	1.33 (1.21)	1.48 (1.20)	<0.001
LDL-c (mean (SD))		112.11 (35.58)	112.24 (35.77)	110.94 (33.79)	0.303
TC (mean (SD))		189.47 (41.45)	189.77 (41.71)	186.74 (38.94)	0.143
CMI (mean (SD))		0.72 (0.99)	0.71 (0.98)	0.88 (1.08)	<0.001

BMI, body mass index; WC, waist circumference; ALT, alanine aminotransferase; AST, aspartate aminotransferase; GGT, gamma-glutamyl transferase; BUN, blood urea nitrogen; Scr, serum creatinine; SUA, serum uric acid; eGFR, estimated glomerular filtration rate; FPG, fasting plasma glucose; HbA1c, glycated hemoglobin A1c; HDL-c, high-density lipoprotein cholesterol; TG, triglyceride; LDL-c, low-density lipoprotein cholesterol; TC, total cholesterol; CMI, cardiometabolic index.

Baseline characteristics including age, gender, race, annual household income, education level, physical activity level, smoking status, Diabetes prevalence, Hypertension prevalence, Cardiovascular disease prevalence, BMI (kg/m^2^), Height (cm), WC (cm), ALT (U/L), AST (U/L), GGT (U/L), albumin (g/L), BUN (mmol/L), Scr (umol/L), SUA (umol/L), eGFR (ml/min/1.73m^2^), FPG (mmol/L), HbA1c (%), HDL-c (mmol/L), TG (mmol/L), LDL-c (mg/dl), TC (mg/dl), and CMI (cardiometabolic index).

### 3.2 Clinical features of the participants according to the tertiles of CMI

**Figure 1:**
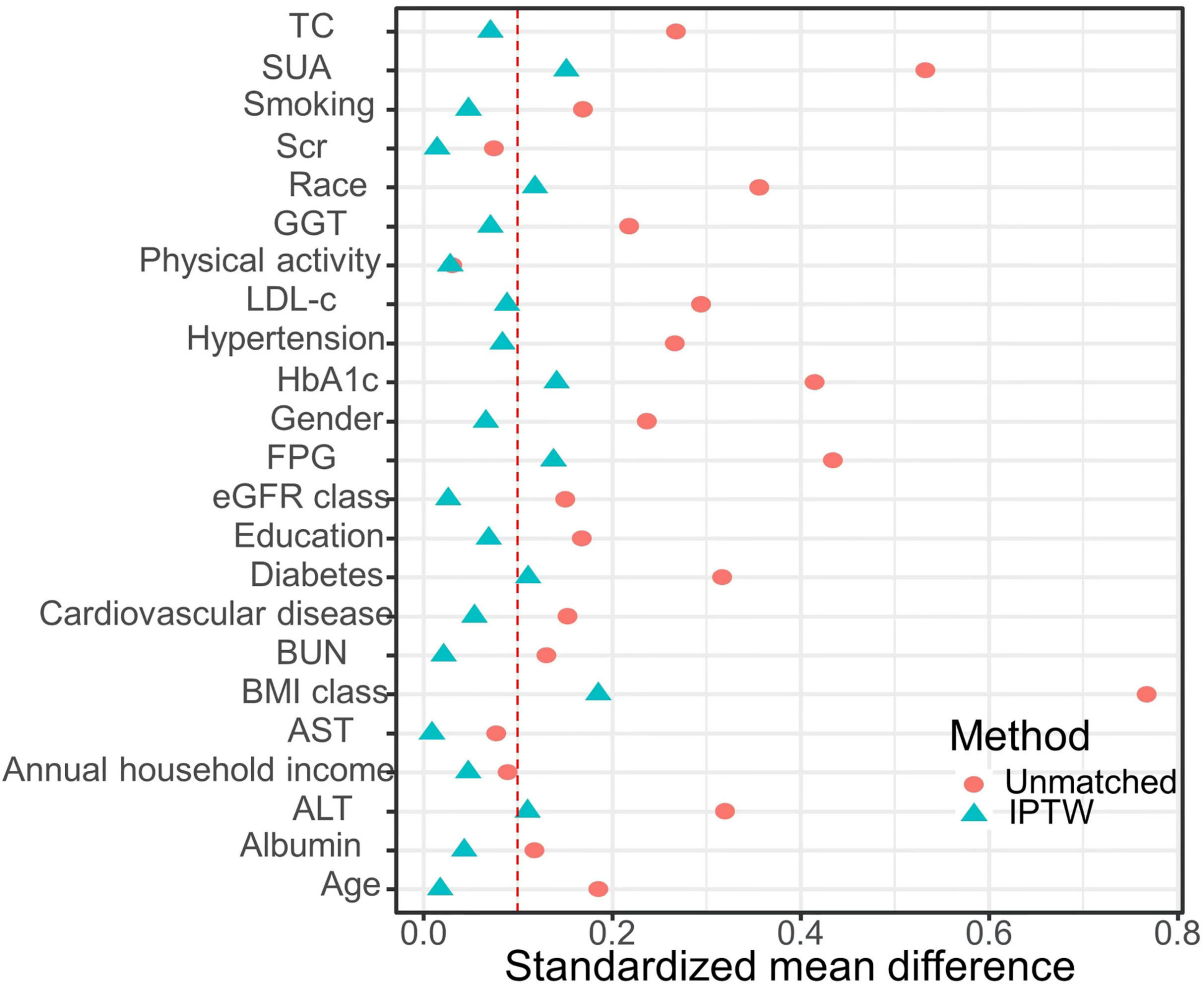
Standardized mean difference between the unmatched model and the IPTW-adjusted model: The red vertical dotted line represents the threshold for acceptable balance, with a standardized mean difference (SMD) < 0.1 indicating a better equilibrium.

Based on the CMI levels, participants were categorized into three groups: tertile I (CMI ≤ 0.332), tertile II (0.332 < CMI ≤ 0.693), and tertile III (CMI > 0.693) (**Table 2**). Compared with the tertile I of CMI, tertile II and tertile III had a higher percentage in male proportion, smoking rates, diabetes prevalence, hypertension prevalence, cardiovascular disease prevalence, and higher levels of age, BMI, height, WC, ALT, AST, GGT, FPG, HbA1c, TG, TC, LDL-c, BUN, Scr, and SUA (*P* < 0.01). Conversely, it had a lower level of HDL-c, eGFR, albumin, education level, and annual household income (*P* < 0.01). Upon IPTW adjustment, most baseline characteristics were well balanced among three groups (SMD < 0.1), except for race, ALT, SUA, BMI, WC, FPG, HbA1c, HDL-c, TG, and the prevalence of diabetes (**Table 2** and **Figure 1**).

**Table 2:** Baseline characteristics of the study population according to the tertiles of CMI.

Characteristics	Unmatched						IPTW				
	level	Tertile I	Tertile II	Tertile III	*P*	SMD	Tertile I	Tertile II	Tertile III	*P*	SMD
N		3059	3059	3059			7738.7	8133.9	7663.4		
Age (mean (SD))		46.63 (18.25)	50.78 (17.36)	51.47 (16.12)	<0.001	0.185	49.78 (17.74)	49.97 (17.41)	50.25 (16.86)	0.737	0.018
Age class (%)	<60	2176 (71.1)	1965 (64.2)	1980 (64.7)	<0.001	0.099	5038.1 (65.1)	5367.3 (66.0)	5069.5 (66.2)	0.755	0.015
	≥60	883 (28.9)	1094 (35.8)	1079 (35.3)			2700.6 (34.9)	2766.6 (34.0)	2593.9 (33.8)		
Gender (%)	Female	1835 (60.0)	1570 (51.3)	1298 (42.4)	<0.001	0.237	4098.4 (53.0)	4248.9 (52.2)	3679.9 (48.0)	0.005	0.066
	Male	1224 (40.0)	1489 (48.7)	1761 (57.6)			3640.3 (47.0)	3885.1 (47.8)	3983.6 (52.0)		
Race (%)	Mexican American	259 (8.5)	443 (14.5)	539 (17.6)	<0.001	0.356	909.1 (11.7)	1122.2 (13.8)	1139.3 (14.9)	0.001	0.118
	Non-Hispanic Black	898 (29.4)	673 (22.0)	377 (12.3)			1809.8 (23.4)	1718.7 (21.1)	1371.9 (17.9)		
	Non-Hispanic White	1088 (35.6)	1111 (36.3)	1284 (42.0)			2875.9 (37.2)	3062.3 (37.6)	2966.9 (38.7)		
	Other Hispanic	232 (7.6)	358 (11.7)	401 (13.1)			753.7 (9.7)	913.3 (11.2)	932.7 (12.2)		
	Other Races	582 (19.0)	474 (15.5)	458 (15.0)			1390.2 (18.0)	1317.4 (16.2)	1252.6 (16.3)		
Annual household income (%)	Above $20,000	2568 (83.9)	2440 (79.8)	2409 (78.8)	<0.001	0.089	6414.5 (82.9)	6531.0 (80.3)	6141.4 (80.1)	0.044	0.047
	Under $20,000	491 (16.1)	619 (20.2)	650 (21.2)			1324.2 (17.1)	1602.9 (19.7)	1522.1 (19.9)		
Education level (%)	Below high school	1133 (37.0)	1392 (45.5)	1512 (49.4)	<0.001	0.168	3238.3 (41.8)	3589.3 (44.1)	3601.9 (47.0)	0.006	0.069
	Above high school	1926 (63.0)	1667 (54.5)	1547 (50.6)			4500.4 (58.2)	4544.6 (55.9)	4061.6 (53.0)		
Physical activity level (%)	Low	1777 (58.1)	1822 (59.6)	1782 (58.3)	0.57	0.03	4496.8 (58.1)	4827.3 (59.3)	4529.5 (59.1)	0.768	0.028
	Moderate	663 (21.7)	644 (21.1)	638 (20.9)			1613.3 (20.8)	1730.3 (21.3)	1598.9 (20.9)		
	High	619 (20.2)	593 (19.4)	639 (20.9)			1628.6 (21.0)	1576.3 (19.4)	1535.0 (20.0)		
Smoking status (%)	No	1910 (62.4)	1762 (57.6)	1528 (50.0)	<0.001	0.169	4438.9 (57.4)	4665.2 (57.4)	4124.0 (53.8)	0.039	0.048
	Yes	1149 (37.6)	1297 (42.4)	1531 (50.0)			3299.8 (42.6)	3468.7 (42.6)	3539.4 (46.2)		
Diabetes (%)	No	2876 (94.0)	2642 (86.4)	2388 (78.1)	<0.001	0.317	6951.4 (89.8)	7024.7 (86.4)	6456.5 (84.3)	<0.001	0.111
	Yes	183 (6.0)	417 (13.6)	671 (21.9)			787.3 (10.2)	1109.2 (13.6)	1207.0 (15.7)		
Hypertension (%)	No	2226 (72.8)	1915 (62.6)	1646 (53.8)	<0.001	0.266	5115.9 (66.1)	5146.2 (63.3)	4601.4 (60.0)	0.001	0.084
	Yes	833 (27.2)	1144 (37.4)	1413 (46.2)			2622.8 (33.9)	2987.8 (36.7)	3062.0 (40.0)		
Cardiovascular disease (%)	No	2839 (92.8)	2714 (88.7)	2624 (85.8)	<0.001	0.153	7025.1 (90.8)	7249.2 (89.1)	6766.1 (88.3)	0.04	0.054
	Yes	220 (7.2)	345 (11.3)	435 (14.2)			713.6 (9.2)	884.8 (10.9)	897.4 (11.7)		
BMI class (%)	Normal weight	1623 (53.1)	743 (24.3)	307 (10.0)	<0.001	0.767	2588.0 (33.4)	2301.1 (28.3)	1712.4 (22.3)	<0.001	0.186
	Overweight	894 (29.2)	1124 (36.7)	956 (31.3)			2637.3 (34.1)	2735.8 (33.6)	2621.5 (34.2)		
	Obese	542 (17.7)	1192 (39.0)	1796 (58.7)			2513.4 (32.5)	3097.0 (38.1)	3329.6 (43.4)		
Height (mean (SD))		166.60 (9.44)	166.90 (10.14)	167.43 (10.27)	0.004	0.056	167.08 (9.63)	166.94 (10.09)	166.65 (10.26)	0.433	0.029
WC (mean (SD))		89.42 (13.56)	100.50 (14.53)	108.91 (15.67)	<0.001	0.892	95.29 (14.61)	99.65 (14.83)	103.82 (16.01)	<0.001	0.374
ALT (mean (SD))		20.73 (16.48)	23.42 (15.18)	29.00 (18.97)	<0.001	0.32	22.55 (15.82)	23.69 (15.83)	25.24 (16.87)	<0.001	0.11
AST (mean (SD))		23.92 (16.31)	23.93 (19.09)	26.12 (20.40)	<0.001	0.077	24.20 (14.46)	24.25 (20.19)	24.43 (17.84)	0.863	0.009
GGT (mean (SD))		23.93 (38.82)	27.82 (31.96)	36.52 (41.85)	<0.001	0.218	26.57 (34.53)	28.13 (32.44)	30.28 (36.33)	0.001	0.071
Albumin (mean (SD))		42.35 (3.46)	41.88 (3.48)	41.73 (3.54)	<0.001	0.117	42.07 (3.40)	42.00 (3.47)	41.85 (3.48)	0.132	0.043
BUN (mean (SD))		4.76 (1.86)	4.92 (2.03)	5.17 (2.36)	<0.001	0.13	4.91 (1.91)	4.90 (1.98)	4.96 (2.12)	0.51	0.021
Scr (mean (SD))		76.37 (44.87)	77.99 (28.43)	80.66 (38.86)	<0.001	0.075	77.35 (38.56)	77.27 (26.40)	77.97 (32.51)	0.642	0.014
SUA (mean (SD))		293.44 (74.22)	326.40 (80.81)	358.51 (88.86)	<0.001	0.533	316.22 (80.56)	324.74 (82.05)	334.98 (84.88)	<0.001	0.151
eGFR class (%)	<60	159 (5.2)	229 (7.5)	283 (9.3)	<0.001	0.15	489.5 (6.3)	577.0 (7.1)	559.3 (7.3)	0.782	0.026
	60~90	822 (26.9)	968 (31.6)	998 (32.6)			2394.4 (30.9)	2496.4 (30.7)	2356.1 (30.7)		
	≥90	2078 (67.9)	1862 (60.9)	1778 (58.1)			4854.8 (62.7)	5060.4 (62.2)	4748.1 (62.0)		
FPG (mean (SD))		5.55 (1.15)	6.03 (1.80)	6.83 (2.62)	<0.001	0.434	5.88 (1.57)	6.03 (1.79)	6.26 (2.07)	<0.001	0.138
HbA1c (mean (SD))		5.49 (0.69)	5.79 (1.06)	6.17 (1.43)	<0.001	0.415	5.67 (0.89)	5.77 (1.05)	5.89 (1.17)	<0.001	0.141
HDL-c (mean (SD))		1.74 (0.42)	1.37 (0.27)	1.08 (0.23)	<0.001	1.37	1.71 (0.40)	1.41 (0.29)	1.09 (0.22)	<0.001	1.36
TG (mean (SD))		0.66 (0.21)	1.11 (0.29)	2.27 (1.70)	<0.001	1.35	0.67 (0.20)	1.15 (0.31)	2.09 (1.29)	<0.001	1.464
LDL-c (mean (SD))		102.78 (31.04)	115.72 (35.46)	117.81 (37.30)	<0.001	0.295	109.10 (33.92)	113.46 (34.99)	113.70 (35.75)	<0.001	0.088
TC (mean (SD))		181.82 (37.21)	188.26 (40.65)	198.33 (44.50)	<0.001	0.268	187.33 (39.86)	188.25 (39.90)	191.66 (41.73)	0.003	0.071
CMI (mean (SD))		0.21 (0.07)	0.49 (0.10)	1.46 (1.44)	<0.001	1.777	0.23 (0.07)	0.49 (0.10)	1.27 (1.09)	<0.001	1.781
Kidney stone (%)	No	2864 (93.6)	2728 (89.2)	2677 (87.5)	<0.001	–	7147.4 (92.4)	7253.6 (89.2)	6835.1 (89.2)	<0.001	0.073
	Yes	195 (6.4)	331 (10.8)	382 (12.5)		–	591.3 (7.6)	880.4 (10.8)	828.3 (10.8)	–	–

Baseline characteristics including age, gender, race, annual household income, education level, physical activity level, smoking status, Diabetes prevalence, Hypertension prevalence, Cardiovascular disease prevalence, BMI class, Height (cm), WC (cm), ALT (U/L), AST (U/L), GGT (U/L), albumin (g/L), BUN (mmol/L), Scr (umol/L), SUA (umol/L), eGFR (ml/min/1.73m^2^).

FPG (mmol/L), HbA1c (%), HDL-c (mmol/L), TG (mmol/L), LDL-c (mg/dl), TC (mg/dl), and CMI (cardiometabolic index).

### 3.3 Association between CMI and kidney stones

**Figure 2:**
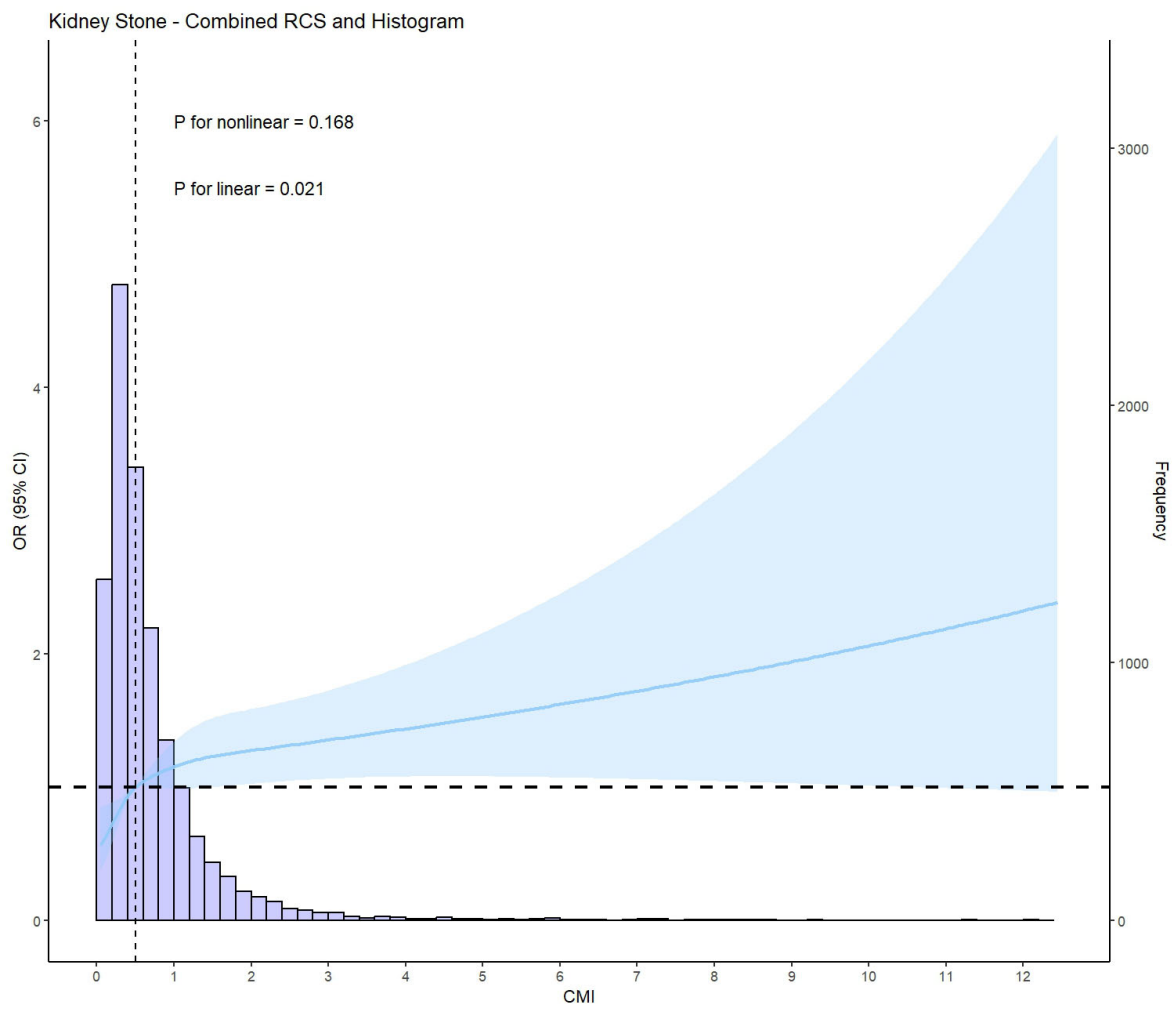
Restricted cubic spline plot (RCS) of CMI and kidney stone (based on model 3, adjusted for age, gender, race, annual household income, education level, physical activity level, smoking status, ALT(U/L), AST(U/L), GGT(U/L), albumin(g/L), BUN(mmol/L), Scr(umol/L), SUA(umol/L), eGFR class, BMI class, FPG(mmol/L), HbA1c(%), LDL-c(mg/dl), TC(mg/dl), Cardiovascular disease prevalence, Diabetes prevalence, and Hypertension prevalence).

Logistic regression analysis showed a positive association between CMI and kidney stones in model 3, after adjusting for multiple covariates (OR: 1.30, 95%CI: 1.04 to 1.61, *P* = 0.021) (**Table 3**). According to the IPTW-multivariable logistic analysis, a similar result was observed in model 3 after adjusting for multiple covariates (OR: 1.31, 95%CI: 1.17 to 1.47, *P* < 0.001). Furthermore, RCS analysis indicated a linear relationship between CMI levels and kidney stone risk (*P* for nonlinear = 0.168, *P* for linear = 0.021) (**Figure 2**).

**Table 3:** Logistic regression analysis results of CMI and kidney stone.

CMI class	Unmatched OR (95%CI), *P* value			IPTW-adjusted OR (95%CI), *P* value		
	Non-adjusted model 1	Adjusted model 2	Adjusted model 3	Non-adjusted model 1	Adjusted model 2	Adjusted model 3
Tertile I	1.00	1.00	1.00	1.00	1.00	1.00
Tertile II	1.78 (1.48, 2.15), <0.001	1.59 (1.32, 1.92), <0.001	1.31 (1.07, 1.60), 0.009	1.47 (1.32, 1.64), <0.001	1.45 (1.30, 1.62), <0.001	1.39 (1.24, 1.56), <0.001
Tertile III	2.10 (1.75, 2.51), <0.001	1.73 (1.44, 2.09), <0.001	1.30 (1.04, 1.61), 0.021	1.46 (1.31, 1.64), <0.001	1.42 (1.27, 1.58), <0.001	1.31 (1.17, 1.47), <0.001
*P*_trend_	<0.001	<0.001	<0.001	<0.001	<0.001	<0.001

model 1 was unadjusted;

model 2 was adjusted for age, gender, and race;

model 3 was adjusted for age, gender, race, annual household income, education level, physical activity level, smoking status, ALT (U/L), AST (U/L), GGT (U/L), albumin (g/L), BUN (mmol/L), Scr (umol/L), SUA (umol/L), eGFR class, BMI class, FPG (mmol/L), HbA1c (%), LDL-c (mg/dl), TC (mg/dl), Cardiovascular disease prevalence, Diabetes prevalence, and Hypertension prevalence.

OR, odds ratio; CI, confidence interval.

### 3.4 Subgroup analysis

**Figure 3:**
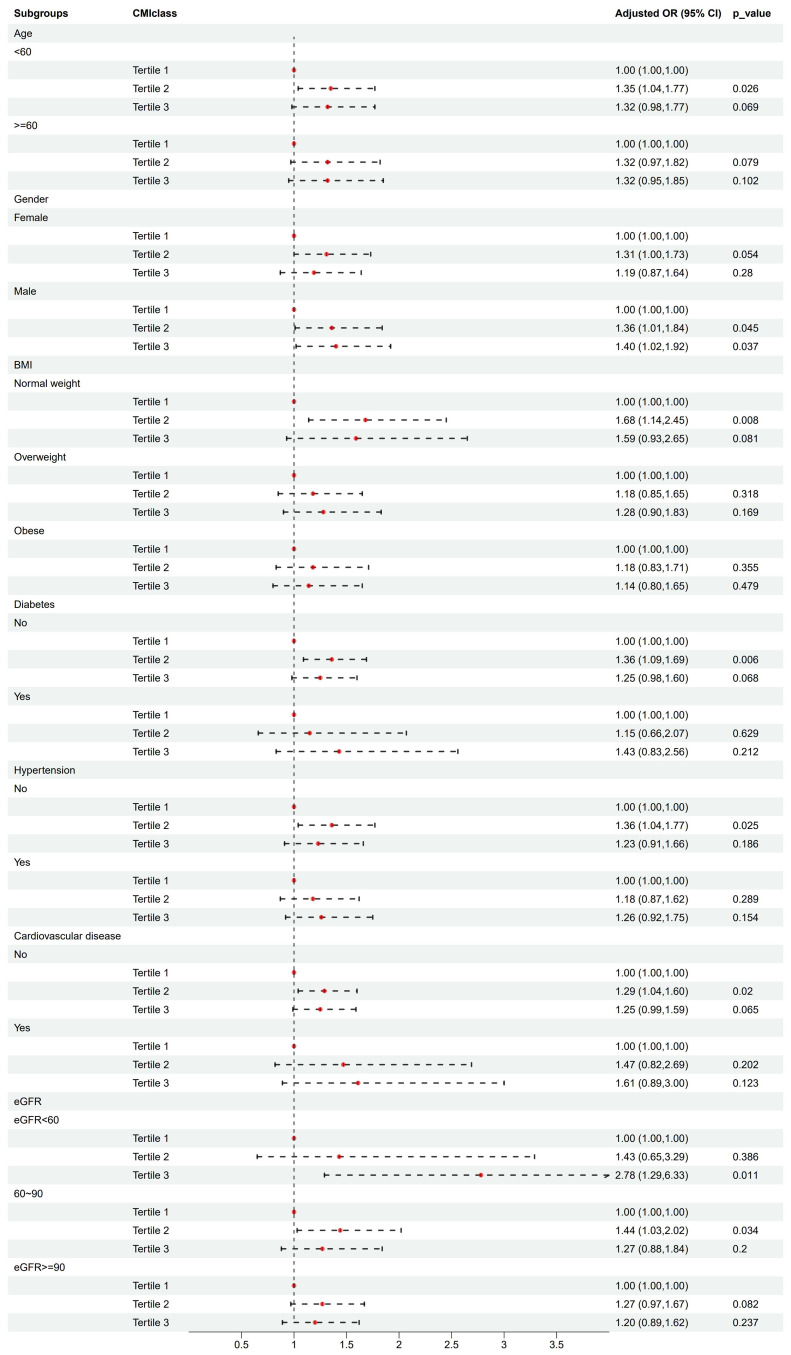
Association analysis of CMI with kidney stones in different subgroups.

Subgroup analysis stratified by age (< 60/≥ 60 years), gender (female/male), BMI (normal weight/overweight/obese), diabetes (yes/no), hypertension (yes/no), cardiovascular disease (yes/no), and eGFR (< 60/60-90/≥ 90 ml/min/1.73m^2^) were used to further assess the relationship between CMI levels and kidney stone risk. According to the IPTW-multivariate adjusted model, the relationship between CMI levels and kidney stone risk was stable by subgroups (**Table 4** and **Figure 3**).

**Table 4:** Association of CMI and kidney stone in different subgroups.

Subgroup	Unmatched OR (95%CI), *P* value			IPTW-adjusted OR (95%CI), *P* value		
	CMI ≤ 0.332 (Tertile I)	0.332<CMI ≤ 0.693 (Tertile II)	CMI>0.693 (Tertile III)	CMI ≤ 0.332 (Tertile I)	0.332<CMI ≤ 0.693 (Tertile II)	CMI>0.693 (Tertile III)
Age						
<60	1 (Ref.)	1.35 (1.04,1.77), 0.026	1.32 (0.98, 1.77), 0.069	1 (Ref.)	1.37 (1.18,1.59), <0.001	1.27 (1.09.1.48), 0.003
≥60	1 (Ref.)	1.32 (0.97,1.82), 0.079	1.32 (0.95,1.85), 0.102	1 (Ref.)	1.47 (1.24,1.75), <0.001	1.43 (1.20,1.70), <0.001
Gender						
Female	1 (Ref.)	1.31 (1.00, 1.73), 0.054	1.19 (0.87, 1.64), 0.280	1 (Ref.)	1.35 (1.15,1.58), <0.001	1.18 (1.00,1.39), <0.001
Male	1 (Ref.)	1.36 (1.01, 1.84), 0.045	1.40 (1.02, 1.92), 0.037	1 (Ref.)	1.49 (1.27,1.76), <0.001	1.51 (1.29,1.78), <0.001
BMI						
Normal weight	1 (Ref.)	1.68 (1.14, 2.45), 0.008	1.59 (0.93, 2.65), 0.081	1 (Ref.)	1.59 (1.25,2.01), <0.001	1.59 (1.23,2.05), <0.001
Overweight	1 (Ref.)	1.18 (0.85, 1.65), 0.318	1.28 (0.90, 1.83), 0.169	1 (Ref.)	1.23 (1.02,1.48), 0.033	1.18 (0.97,1.42), 0.090
Obese	1 (Ref.)	1.18 (0.83, 1.71), 0.355	1.14 (0.80, 1.65), 0.479	1 (Ref.)	1.46 (1.23,1.75), <0.001	1.34 (1.12,1.60), 0.001
Diabetes						
No	1 (Ref.)	1.36 (1.09, 1.69), 0.006	1.25 (0.98, 1.60), 0.068	1 (Ref.)	1.43 (1.26,1.61), <0.001	1.30 (1.15,1.48), <0.001
Yes	1 (Ref.)	1.15 (0.66, 2.07), 0.629	1.43 (0.83, 2.56), 0.212	1 (Ref.)	1.32 (1.00,1.75), 0.055	1.41 (1.07,1.86), 0.014
Hypertension						
No	1 (Ref.)	1.36 (1.04, 1.77), 0.025	1.23 (0.91, 1.66), 0.186	1 (Ref.)	1.47 (1.26,1.71), <0.001	1.25 (1.06,1.46), 0.007
Yes	1 (Ref.)	1.18 (0.87, 1.62), 0.289	1.26 (0.92, 1.75), 0.154	1 (Ref.)	1.29 (1.09,1.52), 0.004	1.36 (1.15,1.61), <0.001
Cardiovascular disease						
No	1 (Ref.)	1.29 (1.04, 1.60), 0.020	1.25 (0.99, 1.59), 0.065	1 (Ref.)	1.33 (1.18,1.49), <0.001	1.22 (1.08,1.38), 0.001
Yes	1 (Ref.)	1.47 (0.82, 2.69), 0.202	1.61 (0.89, 3.00), 0.123	1 (Ref.)	1.91 (1.37,2.69), <0.001	2.08 (1.50,2.93), <0.001
eGFR						
<60	1 (Ref.)	1.43 (0.65, 3.29), 0.386	2.78 (1.29, 6.33), 0.011	1 (Ref.)	1.98 (1.22,3.26), 0.006	3.90 (2.49,6.27), <0.001
60~90	1 (Ref.)	1.44 (1.03, 2.02), 0.034	1.27 (0.88, 1.84), 0.200	1 (Ref.)	1.46 (1.21,1.76), <0.001	1.15 (0.94,1.40), 0.172
≥90	1 (Ref.)	1.27 (0.97, 1.67), 0.082	1.20 (0.89, 1.62), 0.237	1 (Ref.)	1.34 (1.15,1.56), <0.001	1.26 (1.08,1.47), 0.003

## 4 Discussion

To our knowledge, this is the first population-based study to investigate the relationship between CMI level and kidney stone risk. Our study revealed that CMI level was an independent risk factor for kidney stone risk.

The growing interest in CMI stems from its novel combination of TG/HDL-c ratio and WHtR, offering a unified measure that captures aspects of dyslipidemia and central obesity (25). Prior studies implicated CMI in various metabolic disorders, such as atherosclerosis, cardiovascular disease, non-alcoholic fatty liver disease, and diabetes (42, 43). Our findings indicated a significantly higher CMI level among individuals with a history of kidney stones. Furthermore, CMI elevation was associated with a decline in hepatic and renal function, and an increase in BMI, blood glucose, and lipid levels. Current research has linked the incidence of kidney stones to metabolic factors like dyslipidemia, abdominal obesity, hypertension, and diabetes (11). Lee and colleagues identified a correlation between stone risk and several obesity-related measures, such as BMI, WC, WHtR, waist-to-hip ratio, abdominal volume index, and body roundness index (10). In a community cohort study of 121,579 individuals, a significant association was found between kidney stones and metabolic syndrome, with a higher risk of developing kidney stones in participants with metabolic syndrome at baseline (11). Novel indices for assessing visceral obesity, such as the Visceral Adiposity Index and weight-adjusted waist index, have been demonstrated to correlate with the risk of kidney stones, offering a novel perspective in the identification of potential risk factors for kidney stone development (13, 44). Our study effectively integrated the characters of visceral obesity and dyslipidemia, and used IPTW to equilibrate intergroup differences, thereby enhancing the stability and reliability of the analysis results. CMI is a promising tool for identifying kidney stone risk, offering significant clinical and translational value. Firstly, CMI enables rapid and cost-effective assessment of patients’ metabolic health, helping to identify individuals with high risk for kidney stones. This is particularly crucial in primary care settings and resource-limited healthcare systems (45, 46). Secondly, integrating CMI into the risk assessment process for kidney stones may facilitate the implementation of nutritional guidance, exercise programs, and other lifestyle interventions, which can help improve long-term health outcomes for high-risk groups.

The interconnections between obesity, metabolic syndrome, and dyslipidemia underscored the systemic nature of kidney stone disease. The precise mechanisms by which CMI influences stone formation are yet to be fully delineated. Our findings suggested that the dysregulated lipid metabolism associated with elevated CMI levels may contribute to this process. Oxidative stress plays a key role in metabolic disorder onset, with escalating levels of it in obesity and dyslipidemia (47). Hyperlipidemia, through oxidative stress, can trigger glomerulosclerosis and tubulointerstitial damage, serving as a critical precursor for kidney stone formation (48). Emerging evidence from previous studies suggested a significant association between serum lipids and urinary metabolite profiles, which may influence nephrolithiasis stone composition (49, 50). Dyslipidemia, particularly hypertriglyceridemia, has been identified as a contributing factor in the formation and recurrence of kidney stones (51). Notably, patients with low HDL-c or elevated TG levels demonstrate markedly increased urinary sodium, oxalate, and uric acid concentrations, along with decreased urinary pH values (52). The use of lipid-lowering medications, notably atorvastatin, has been observed to significantly alter urinary biochemical profiles (53). Metabolic syndrome, often characterized by central adiposity, was implicated in kidney stone pathology due to its association with altered urinary excretion patterns of lithogenic substances like calcium, uric acid, oxalate, and citrate. Furthermore, oxidative stress induced by visceral fat may contribute to renal impairment through increased production of inflammatory cytokines like interleukin-6, C-reactive protein, and reactive oxygen species, which could facilitate stone formation (54).

One of the primary strengths of our study lies in its large sample size, which is representative of the general U.S. adult population. The comprehensive adjustment for a multitude of potential confounders ensured the robustness of the CMI-nephrolithiasis association. Nevertheless, our study’s interpretive potential is tempered by its inherent limitations. The cross-sectional design may hinder our ability to establish causality, and our reliance on self-reported history of kidney stones may introduce bias. However, the credibility of the NHANES database in reflecting public health concerns in the U.S., including the epidemiology of stone disease, substantiates our methodology (29–31, 55). The lack of detailed typological data on kidney stones in our dataset may preclude a more nuanced stone composition analysis. Although full adjustments were made for common confounding factors, potential residual confounders such as lipid-lowering medications or specific dietary habits were not accounted for, which may introduce bias. We recommend prospective, multi-center studies to further elucidate and confirm the prognostic utility of CMI in kidney stone risk evaluation.

## 5 Conclusion

In conclusion, our study found a significant association between CMI level and the risk of kidney stones after adjusting for potential confounders. CMI could be utilized as an effective tool for identifying individuals at high risk of kidney stones during routine health examinations.

## Data Availability

The datasets presented in this study can be found in online repositories. The names of the repository/repositories and accession number(s) can be found in the article/**Supplementary Material**.

## Supplementary material

Supplementary Figure 1 Distribution of CMI data.

Supplementary Figure 2 Presentation of the Restricted cubic spline (RCS) analysis of the complete dataset, with the x-axis displaying the full range of CMI values (based on model 3, adjusted for age, gender, race, annual household income, education level, physical activity level, smoking status, ALT (U/L), AST (U/L), GGT (U/L), albumin (g/L), BUN (mmol/L), Scr (umol/L), SUA (umol/L), eGFR class, BMI class, FPG (mmol/L), HbA1c (%), LDL-c (mg/dl), TC(mg/dl), Cardiovascular disease prevalence, Diabetes prevalence, and Hypertension prevalence).
