# Supplementary figures and images for: Association between cardiometabolic index and kidney stone from NHANES: a population-based study
Source: Front Endocrinol (Lausanne). 2024 Oct 9;15:1408781. doi: 10.3389/fendo.2024.1408781 (PMC11498271; doi:10.3389/fendo.2024.1408781)

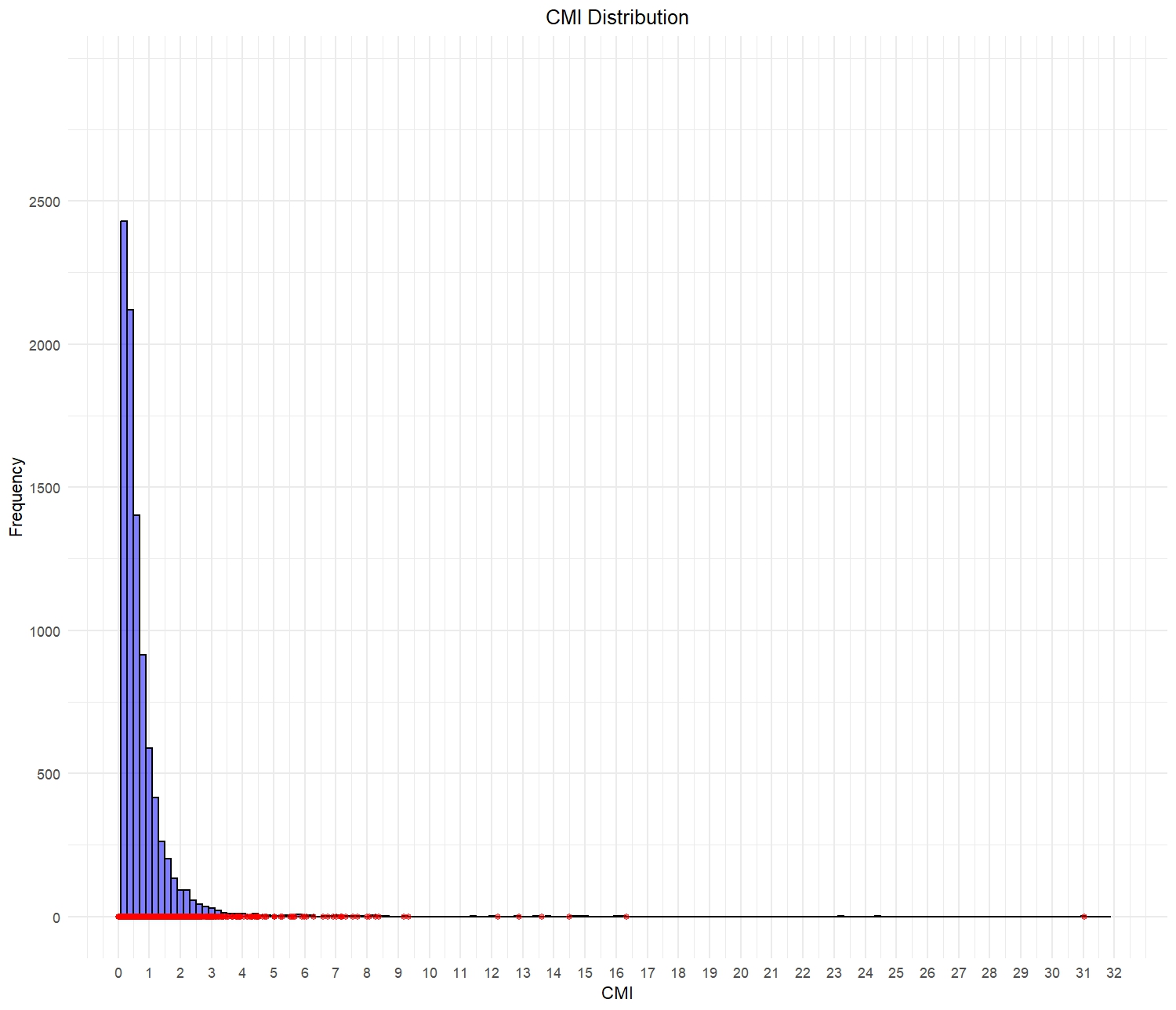

Supplement: Supplementary Figure 1 — Distribution of CMI data. [file Image1.jpeg]

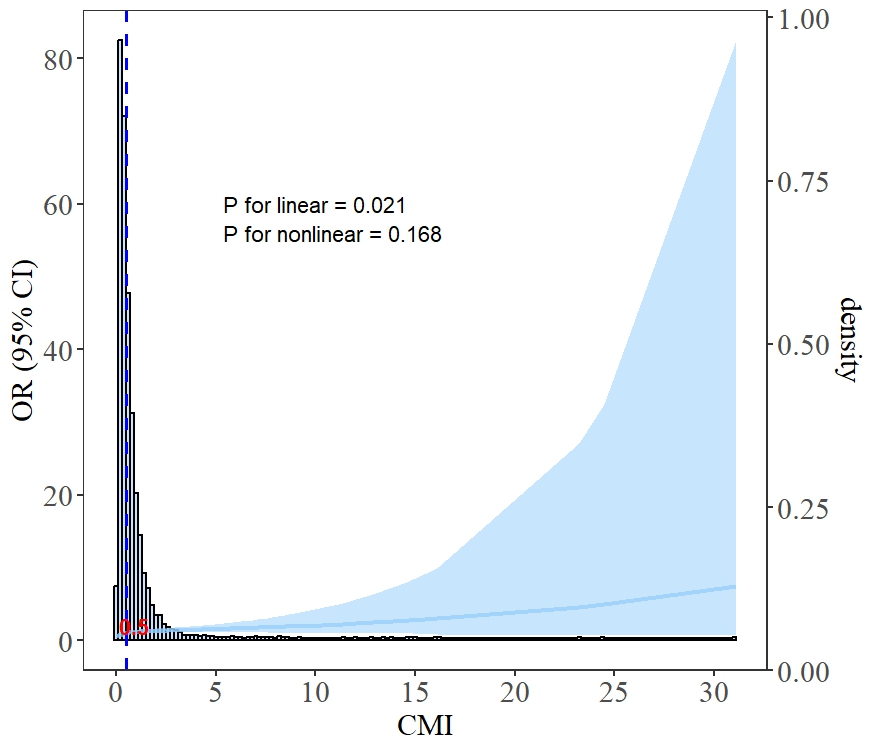

Supplement: Supplementary Figure 2 — Presentation of the Restricted cubic spline (RCS) analysis of the complete dataset, with the x-axis displaying the full range of CMI values (based on model 3, adjusted for age, gender, race, annual household income, education level, physical activity level, smoking status, ALT (U/L), AST (U/L), GGT (U/L), albumin (g/L), BUN (mmol/L), Scr (umol/L), SUA (umol/L), eGFR class, BMI class, FPG (mmol/L), HbA1c (%), LDL-c (mg/dl), TC(mg/dl), Cardiovascular disease prevalence, Diabetes prevalence, and Hypertension prevalence). [file Image2.jpeg]
